# Health Concerns of Iranian Adolescents: Protocol for a Mixed Methods Study

**DOI:** 10.5812/ircmj.6715

**Published:** 2014-06-05

**Authors:** Azam Baheiraei, Elham Khoori, Fazlollah Ahmadi, Abbas Rahimi Foroushani, Fazlollah Ghofranipour

**Affiliations:** 1 Department of Reproductive Health, School of Nursing and Midwifery, Tehran University of Medical Sciences, Tehran, IR Iran; 2 Community Based Participatory Research Center, Iranian Institute for Reduction of High-Risk Behaviors, Tehran University of Medical Sciences, Tehran, IR Iran; 3 Department of Nursing, Faculty of Medical Sciences, Tarbiat Modares University, Tehran, IR Iran; 4 Department of Epidemiology and Biostatistics, School of Public Health, Tehran University of Medical Sciences, Tehran, IR Iran; 5 Department of Health Education, School of Medical Sciences, Tarbiat Modares University, Tehran, IR Iran

**Keywords:** Adolescents, Iran, Health information, Health Education

## Abstract

**Background:**

Adolescents have particular health and developmental needs that suggest they should neither be treated as older children nor younger adults.

**Objectives:**

The aim of this paper is to report the protocol for a mixed methods study that set out to investigate the health concerns of Iranian adolescents and their sources of health information with the goal of identifying suitable strategies to address their health concerns.

**Materials and Methods:**

This mixed methods study consists of an explanatory sequential design to be conducted in two phases. The first phase was a population-based cross-sectional survey of 915, 14-18 year old adolescents who were selected by stratified cluster random sampling method from the 22 main municipal sectors of Tehran, Iran. They completed a series of self-administered questionnaires which were analyzed using quantitative approaches. The second phase was a qualitative study in which adolescents were selected using purposeful sampling for individual in-depth semi-structured interviews on the basis of the quantitative findings from the first phase. These data, together with a literature review and data obtained through nominal group technique, would then be used to in the development of strategies to reduce adolescents’ health concerns.

**Results:**

The findings of this mixed methods sequential explanatory study are expected to provide unique information about the health concerns of Iranian adolescents and their sources of information, which to date have received little attention.

**Conclusions:**

These data will provide a rich source of information that can be used by intervention programs, health professionals and policy makers in addressing the health concerns of adolescents, with the goal of facilitating a successful passage to adult life.

## 1. Background

Adolescence is one of the most significant periods in a person's life. During this period, critical physical, cognitive, psychological, behavioral and social maturational changes take place that can affect the health of young people during adolescence itself as well as into the adult life (1-3). Adolescents have a sense of curiosity about the world around them. A normative and universal aspect of healthy adolescent development is that young people learn to function more autonomously from their families, achieve a stronger sense of independence and have a greater desire for privacy (4, 5). As a result, many of their physical, psychological and social concerns can remain unknown to their families and are instead shared with their peers who are not always reliable sources of health information (5).

The health issues facing young people are distinct from those facing younger children whose health problems is dominated by infectious diseases, and adults whose health problems is dominated by non-communicable diseases. In contrast adolescents across the world experience high rates of injury, sexual and reproductive health problems, bullying and mental disorders and nutritional concerns such as obesity and eating disorders (2). Adolescence is also the time of onset of many behaviors that affect health, such as tobacco and substance use, where the health consequences typically contribute most to the burden of diseases in the adulthood. This complex health burden results in teenagers having special health and development needs, especially in relation to anticipatory guidance (2, 4). Adolescents are interested in obtaining information about many different health concerns and seek answers to many questions (6). Health concerns represent “personal salience of health issues (e.g. events, problems, risks, threats of diseases, etc.), linking motivation and behavior” (7). Health concerns can differ according to age, gender and various periods of life (7).

Notwithstanding the complex developmental challenges facing young people in contemporary society, there is remarkably little information about their particular health needs (2). In order to understand this, it is important that the opinions of adolescents themselves are sought in relation to identifying their health concerns and their sources of information about these. Although studies in other countries could provide some useful data, the extent of political, socio-cultural and religious differences, together with different availability of relevant resources, suggests that each country needs its own research data in order to better understand how the health concerns of young people can be best met (8, 9). This is consistent with the World Health Organization (WHO) notion that national level data is necessary to inform national decision makers in relation to the most relevant policies to support young people (10, 11).

While there has been much progress in primary health care in Iran, the particular health needs of adolescents have been neglected.

## 2. Objectives

The aim of this study was to identify Iranian adolescents’ health concerns as well as their sources of health information. This study also aimed at developing strategies in response to the most common health concerns of adolescents that were identified through this work. The specific objectives were:

1. To identify Iranian adolescents' health concerns by gender and socio-demographic characteristics,
2. To identify Iranian adolescents’ sources of health information about their health concerns, and
3. To offer strategies to address the adolescents’ most common health concerns.

This protocol paper sets out to describe the detailed methods used to identify these concerns in a study that was undertaken in 2011. The results will be presented elsewhere in a later publication. 

## 3. Materials and Methods

This study used a mixed methods approach. It used an explanatory sequential design of the follow up explanation model. This model starts with data collection and analysis of a quantitative phase which is then followed by qualitative data collection and analysis (12). This design is used when qualitative data is valuable to help explain or expand the quantitative findings, especially in the context where further explanation is warranted regarding significant or non-significant differences between groups, surprising associations, or unexpected results and extreme cases (12, 13).

### 3.1. The First Phase, Quantitative Study

The quantitative study was a population-based cross-sectional study to assess adolescents’ health concerns and sources of health information, and to investigate the relationship between health concerns and various socio-demographic characteristics. The source population was every 14-18 year old adolescent living in Tehran. In Iranian culture, children usually live with their parents until marriage, regardless of age (14).

Based upon the definition of the WHO of adolescence [early (10-14 years), middle (15-17 years) and late (18-19 years)], the age category of 14-18 years was identified because for cultural reasons in Iran, asking sensitive questions (e.g. about sexual issues and drug use) in early adolescence would not be deemed appropriate (10).

#### 3.1.1. Sample Size and Sampling Method

The sample size was calculated on the basis of two studies (1, 15) which used 20 and 25 items questionnaires with the range of scores between 0-60 and 0-100, respectively. Based on these two studies, and together with Weiler’s study (16), whose standard deviation of subscales and total inventory means were reported as 11-31, a standard deviation of 31 was selected. The sample size (n = 410) was calculated based on 95% confidence level and a maximum difference of three points between the estimated and true values. As the population to be sampled consisted of 14-18 year old adolescents of Tehran living in families, it is possible that their responses might be clustered by being from the same family. Therefore, in calculating the sample size, the design effect coefficient of two was chosen to increase the sample size to 820 (410 × 2). To account for the potential loss of 10% of the sample, the final sample size was increased to 915 adolescents.

Different regions of Tehran have their own socio-cultural and economic characteristics. Therefore, adolescents were selected using a stratified cluster random sampling method. To increase the accuracy and to adjust for distribution characteristics, the 22 main municipal sectors of Tehran were divided into five arbitrary geographic regions: north, south, west, east and center. By knowing the adolescent population of the 22 municipality districts of Tehran city, samples were obtained in each area according to the population and gender ratio. Within each area, one female high school and one male high school were randomly selected from the list of all high schools. Adolescents whose families lived near the high school were selected for participation in the study, where each family was considered a cluster. Parents were asked to provide informed consent and adolescent participants were asked to express their agreement to take part in the study. The inclusion criteria were:

1. Iranian nationality,
2. Aged 14-18 years,
3. Sufficiently literate to complete written surveys,
4. Resident of Tehran,
5. Single.

Participants were told they would complete the questionnaires anonymously at home, where each questionnaire was coded by number. They were also asked if they could leave a contact number in order that they could be subsequently invited to participate in the qualitative phase.

#### 3.1.2. Scales and Data Collection

The Adolescent Health Concern Inventory-Persian version (AHCI-P) was administered to all participants, together with a questionnaire that included sources of health information and demographic questions. The AHCI was developed by Professor Weiler in the United States of America. Permission was obtained to translate the AHCI from English into Persian. This was professionally undertaken, including back translation to compare with the original version and was then pilot tested with Iranian adolescents. The AHCI comprises three sections: health and health education (Section A); health concerns (Section B); and demographic characteristics (Section C). In this research, only section B was administered, which contains 150 health-related items divided into 12 subscales. These were Disease and Disorders, Substance Use and Abuse, Consumer Health, The Environment, Human Sexuality, Injury Prevention and Control, Personal Health, Social Health, Relationships, Emotional Health, Nutrition, and The Future (16).

Initially, validity and reliability of the questionnaire was examined. The face and content validity were also assessed. Test- re-test reliability was examined by asking 50 participants to repeat the questionnaire within a 2-week interval. Cronbach’s alpha was calculated as a reliability test of internal consistency. The findings from the pilot test indicated that the AHCI-P is both valid and reliable and is a practical tool for assessing the health concerns of adolescents. A detailed description of the psychometric properties of the AHCI in an Iranian population has been published elsewhere (17).

#### 3.1.3. Data Analysis

Data were analyzed using SPSS software. The descriptive statistics included mean, standard deviation, median and frequency. The Kolmogorov-Smirnov test was used to determine if the distribution of scores was normal or not. Non parametric statistics were applied to evaluate the association between the total health concern scores and demographic factors. Finally, to study the effects of statistically significant independent variables on the dependent variable (health concern), multiple regression analysis was undertaken. In addition, to include the effect of cluster sampling in data analysis, STATA 10 was used to find accurate estimates.

### 3.2. The Second Phase, Qualitative Study

The quantitative study was expected to identify the most common health concerns experienced by Iranian adolescent. The qualitative study was then undertaken in order to gain a deeper understanding of these data. In particular, this was undertaken in order to interpret the quantitative findings in terms of better understanding of what meaning these young people attributed to these issues.

#### 3.2.1. Sample Size and Sampling Method

Sample size could not be determined by a prior qualitative research. Rather, sampling continues until data saturation is reached. The sample was selected using purposeful sampling (“the researcher purposely selects participants who have experienced the central phenomenon or the key concept being explored”) (13) using the strategy of maximal variation sampling. This means that adolescents are purposefully selected to be diverse in age, socio-economic, gender, etc. It is expected that their perspectives will similarly echo this difference and offer a high-quality qualitative study which will provide a more complete view of adolescents’ most prevalent health concerns (12).

If the adolescents agreed, they were eligible for inclusion in the qualitative phase of the study. Inclusion criteria were:

1. Previous participation in the quantitative phase;
2. Participants who experienced the most common health concerns identified in the quantitative phase.

#### 3.2.2. Data Collection

Data were collected through individual interviews rather than using focus groups. As it was considered that participants may not feel comfortable expressing sensitive issues in front of others, their deeper views would more likely be obtained using individual interviews (13, 18). The aim was to enquire and explore the experiences of selected adolescents of their most common health concerns and their proposed solutions to address these concerns. Adolescents chose the place for the interview. The semi-structured interviews continued until data saturation was achieved, that is, no new themes were emerged. In addition to the interviewer notes during interviews, each interview was audio-recorded.

### 3.3. Training of the Interviewer

The interviewer was a PhD student who was undertaking this research as her doctoral thesis [corresponding author]. She has significant clinical experience communicating with adolescents in a manner that would be expected to facilitate greater ease in discussing highly sensitive information. She was trained in qualitative interviewing as part of her doctoral training.

### 3.4. Data Analysis

MAXQDA software version 10 was used for the data management. The qualitative data were analyzed using a conventional content analysis approach. The advantage of a conventional approach in qualitative content analysis is the ability to gather data directly from participants without imposing pre-conceived categories and previous theoretical perspectives. In this method, knowledge generated from content analysis is based on the unique views of the participants and is rooted in the text data (19).

In this part of the research, the recordings are listened to several times to establish a general over-view. After this, each audio-taped interview was accurately transcribed and read repeatedly in order to obtain the theme of the adolescent’s story, as one would read a novel. The text was coded and codes were put in sentences or important paragraphs until the categories formed and finally, themes emerged.

In order to establish credibility of the data in a qualitative study, a method of external checks can be used. In particular, the results of the analysis and a summary of the research is given to others whose opinions and criticisms are sought, in order to identify whether the researcher’s views are consistent with others. To enable such critique, accurate recording of each stage of the research such as the use of tapes, researcher notes, texts and analysis are provided.

## 4. Results

### 4.1. Integration of Quantitative and Qualitative Data

It is not enough to just collect and analyze quantitative and qualitative data. Rather, these different aspects require integration to shape a more comprehensive picture of the results than when they performed alone (13). At this stage, in addition to reviewing the relevant literature on strategies for reducing adolescents’ health concerns, a nominal technique group (NTG) was undertaken comprised of interested professionals to obtain their opinions about strategies to reduce Iranian adolescents’ health concerns. “Nominal Group Technique (NGT) is a brainstorming tool for quality improvement; it is a highly structured small group discussion used to elicit and prioritize a list of answers to a specific question” (20).

## 5. Discussion

This protocol describes the approach that we undertook in using a combination of quantitative and qualitative methods to provide unique information about the health concerns of Iranian adolescents and their sources of health information. It is well appreciated that adolescence is a time where there are dynamic changes in health and wellbeing including sexual and reproductive health, mental health, nutrition, and possible injuries. There are similarly major changes in the social context of adolescence in most parts of the world, including the Middle-East, with dramatic changes in economic wealth, equal access to education for both boys and girls, and gender roles (2). Adolescence is the time when most young people are still supported by their families yet are universally interested in expanding their social world beyond their immediate family to include their peers (2, 4). The extent of these biologically and socially driven changes make it highly relevant that families, schools, communities, health professionals and policy makers understand the health concerns of adolescents (2, 21). The findings of this mixed methods sequential explanatory study will provide basic information about adolescents’ health concerns and their sources of information that hopefully will be useful in informing the span of interested parties in the community.

The main strength of this study is the goal of recruiting a large and diverse sample of Iranian adolescents aged 14-18 years from Tehran. This factor will increase our ability to generalize the findings of the study as representative of urban Iranian adolescents. The combination of both quantitative and qualitative methods enables a deep analysis of the findings. Potential limitations would be a lack of parental consent and/or adolescents’ unwillingness to participate in the study. The other potential limitation would be if, due to the extent of socio-cultural and religious concerns, many of the possible themes for discussion that involve sensitive health concerns may be under-reported.
